# Effect of Electrical Muscle Stimulation and Resistance Exercise Intervention on Physical and Brain Function in Middle-Aged and Older Women

**DOI:** 10.3390/ijerph20010101

**Published:** 2022-12-21

**Authors:** Ngeemasara Thapa, Ja-Gyeong Yang, Seongryu Bae, Gwon-Min Kim, Hye-Jin Park, Hyuntae Park

**Affiliations:** 1 Department of Health Sciences, Graduate School, Dong-A University, Busan 49315, Republic of Korea; 2 Laboratory of Smart Healthcare, Dong-A University, Busan 49315, Republic of Korea; 3 Medical Research Institute, Pusan National University, Busan 49241, Republic of Korea

**Keywords:** electrical muscle stimulation, resistance exercise, sarcopenia, physical function, cognitive function, combined exercise, short-term intervention

## Abstract

This study investigated the effectiveness of electrical muscle stimulation (EMS) with resistance exercise training (ERT) and resistance exercise training (RT) on physical and brain function in middle-aged and older women. Method: Forty-eight participants were randomly allocated into three groups: (i) ERT (*n* = 16), (ii) RT (*n* = 16), and (iii) control group (*n* = 16). The intervention session was 50 min long and performed three times/week for four weeks. The ERT group performed quadriceps setting, straight leg raises, and ankle pump exercises while constantly receiving EMS on their quadriceps muscle on both legs. The RT group performed the same exercise without EMS. Physical function was measured using skeletal muscle mass index (SMI), handgrip strength, gait speed, five times sit-to-stand test (FTSS) and timed up-and-go test (TUG). Brain function was assessed with electroencephalogram measurement of whole brain activity. Results: After four-week intervention, significant improvements were observed in SMI (*p* < 0.01), phase angle (*p* < 0.05), and gait speed (*p* < 0.05) in the ERT group compared to the control group. ERT also increased muscle strength (*p* < 0.05) and mobility in lower limbs as observed in FTSS and TUG tests (*p* < 0.05) at post-intervention compared to the baseline. In the ERT group, significant positive changes were observed in Beta1 band power, Theta band power, and Alpha1 band whole brain connectivity (*p* < 0.005) compared to the control group. Conclusions: Our findings showed that ERT can improve muscle and brain function in middle-aged and older adults during a four-week intervention program whereas significant improvements were not observed with RT. Therefore might be one of the feasible alternative intervention to RT for the prevention of muscle loss whilst improving brain function for middle-aged and older population.

## 1. Introduction

Ageing is accompanied by several changes in the body including skeletal muscle loss. However, the decline in muscle mass starts at middle age [1], which might lead to increased risk of sarcopenia. Sarcopenia is defined by progressive and generalized loss of skeletal muscle mass, strength, stem cells, and physical function, resulting in poor health outcome such as physical disability, diminished quality of life, and eventually death [2,3,4]. The prevalence of sarcopenia, based on definition, is 5–13% in individuals over the age of 60 years and 11–50% over the age of 80 years [5]. The adverse effects of sarcopenia on quality of life, physical disability, and mortality can affect both middle-aged and older-aged adults. In addition to physical effects, epidemiological studies have suggested that sarcopenia further accelerates cognitive impairment [6] and increases the prevalence of mild cognitive impairment (MCI) and dementia [7,8,9]. 

There are several risk factors associated with sarcopenia including age, gender, frailty, comorbidities, diet, nutrition, body mass index (BMI), and physical inactivity [10,11]. These risk factors also overlap with those associated with MCI (age, poor education, depression, comorbidities, low muscle strength, and lack of physical activity) [12,13]. Physical inactivity, in particular, has a strong correlation with loss of muscle mass and strength, suggesting that exercise intervention/workout can be used as a protective factor against prevention and the management of sarcopenia and MCI [5,14]. Resistance exercise training (RT) is primarily recommended as an effective therapeutic strategy to combat sarcopenia [15] as it increases muscle hypertrophy and strength [16] and physical performance in middle-aged and older adults [17,18]. Other forms of exercise such as aerobic training have also been shown to improve mobility, disability [19], and cognitive function [20], resulting in increased quality of life. In a recent meta-analysis, resistance training has been reported to improve muscle mass and strength as well as quality of life in elderly people with sarcopenia better than aerobic training [21]. In addition, six months of high-intensity resistance training has also been reported to help promote better cognition in people with MCI [14]. However, RT induces a substantial amount of fatigue and may induce too much discomfort, especially in older adults [21]. 

Electrical muscle stimulation (EMS) is another form of exercise that does not require the participants to be physically active [22], which could be an alternative to active exercise. EMS has been employed in medical areas such as in patients with chronic obstructive pulmonary disease [23,24] and chronic heart failure [25], where the benefits were perceived in terms of exercise capacity [24], skeletal muscle performance [23,24], and quality of life [23,25]. In healthy sedentary adults, EMS intervention of 8 weeks performed for 60 min, 5 days/week, has been reported to show increase in the quadriceps muscle strength and six-min walking test distance compared to the baseline [26]. Meanwhile, in healthy older adults, EMS has been reported to increase muscle torque, a faster gait speed, increase the diameter of muscle fibers, and other molecules linked with satellite cell differentiation [27]. In the recent scoping review, it was found that very few studies have examined the impact of EMS on quadriceps strength and mass in older adults [28], and it is important to conduct more high-quality RCTs in order to determine whether and to what extent EMS can be used to minimize the deleterious effects of sarcopenia. In addition to physical function, EMS intervention of 40-minute sessions, 5 days per week, for 8 weeks has also been reported to increase brain-derived neurotrophic factor (BDNF) [29]. This evidence suggests that EMS can be used for prevention and treatment of muscle loss as well as have a positive influence on the brain.

Since both exercise and EMS are potentially beneficial for sarcopenia and MCI, we hypothesized that RT in combination with EMS will be more effective than performing RT or EMS alone. A study on subjects with anterior cruciate ligament (ACL), EMS in combination with rehabilitation program, for duration of 4 weeks (5 days/week) was reported to be effective in maintaining and increasing muscle thickness and strength compared to the control group who only performed a rehabilitation program [30]. However, in this study, EMS and rehabilitation exercises were performed separately. Currently, studies comparing the effectiveness of short-term combined EMS with structured exercise training, performed simultaneously, are lacking. Therefore, the purpose of our study was to investigate the effects of short-term use of EMS administered constantly while performing RT (ERT) and single bout RT intervention on physical and brain function in community dwelling middle-aged and older adults. 

## 2. Materials and Methods

### 2.1. Study Design and Sample 

**Figure 1:**
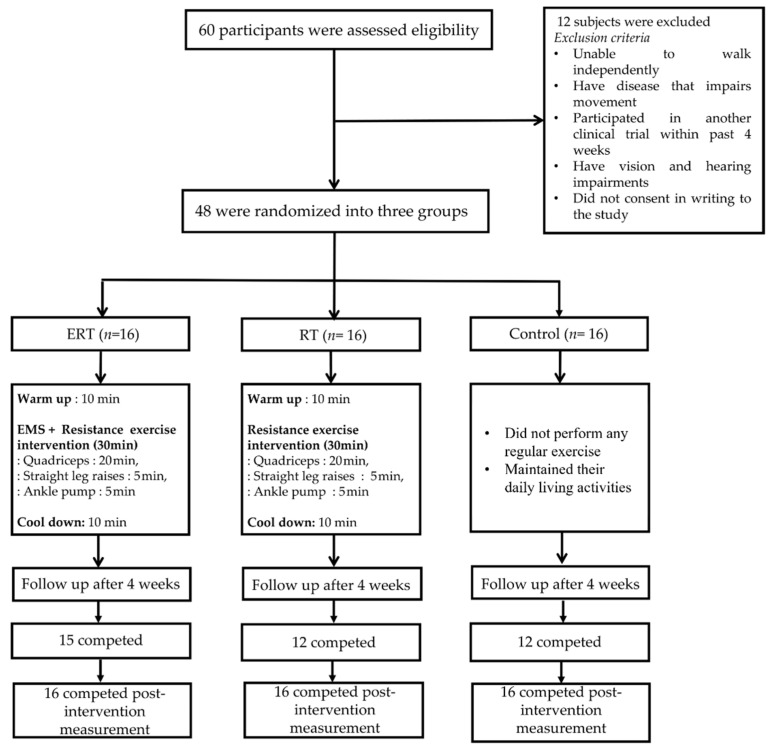
Flow diagram of study participants.

In this randomized control trial (RCT), participants were recruited from Busan Metropolitan City, Korea. Inclusion criteria were female participants between 40 and 85 years of age, able to walk independently, and free of orthopedic disease. Sample size was calculated using G*Power 3.1.9.4 [31]. Based on a previous study on EMS with physical function as outcome variables [32], we calculated a medium effect size of the intervention (Cohen’s f = 0.50). Therefore, using a statistical power of 0.80, alpha of 0.05, and effect size of 0.5, a total sample size of 48 was calculated and allocated to three groups: (i) ERT (*n* = 16), (ii) RT (*n* = 16), and (iii) control (*n* = 16). The study design and exclusion criteria are described in Figure 1. The study procedures were approved by Dong-A University Institution Review Board (IRB No. 2-1040709-AB-N-01-202201-HR-008-02). Informed consent was received from all participants before enrollment in the study. 

### 2.2. Intervention

The ERT consisted of a series of resistance exercises performed by the participants while being administered with EMS (EXOPILL, EXOSYSTEMS, Seoul, Korea) in their quadriceps muscle on both legs. The EMS pads were attached vertically along the midline in the center of the knee and pelvis. The exercise program was conducted using an application provided by the same company. The intervention was performed for 50 min three times/week for four weeks. Each session consisted of warm-up time of 10 min, quadriceps setting for 20 min, straight leg raises performed twice for 5 min each, an ankle pump for 5 min, and cool-down time of 10 min. During the intervention period, participants used the application independently under the supervision of experienced researchers. At the beginning of the intervention, researchers instructed participants on ways to correctly use the exercise application. EMS was administered at frequency = 35 to 70 Hz, pulse duration = 100 μs, pulse period = 50 ms, and size = 150 mm × 240 mm. EMS intensity levels were increased gradually over 4 weeks. The RT group performed the same resistance exercises as the ERT group without using EMS. The control group did not perform any physical exercise but attended seminars on prevention of geriatric disease such as frailty and dementia once a week for four weeks.

### 2.3. Physical Function

Muscle strength was measured with a hand grip strength (HGS) test using a digital hand-held dynamometer (TKK 5101 Grip-D, Takei, Tokyo, Japan). During the HGS test, participants were instructed to stand straight while maintaining their arms slightly apart from their body and hold the dynamometer pointing to the ground. The HGS test was performed twice, and the average value was calculated and used for analysis. At a comfortable walking pace, a 7 m gait speed test was conducted, comprising an acceleration phase of 1.5 m, a 4 m walk (starting and ending points marked on the floor), and a 1.5 m deceleration phase. In five times sit-to-stand test (FTSS), participant’s speed to stand up from a chair as quickly as possible and sit down consecutively for five times was timed. During the test, participants were instructed to cross their arms in front of their chest. Mobility was measured with timed up-and-go (TUG) test. The participants remained seated on a chair before the test started. When a signal was given, participants were required to get up from a chair, walk a distance of 3 m, turn around, walk back to the chair, and sit. Participants were asked to walk at a brisk pace without running. The test was performed twice, and the shortest time was used in analysis.

Anthropometric measurements, such as height and weight, and socio-demographic measures such as age, sex, and education were also acquired. Body composition parameters including body mass index (BMI), skeletal muscle mass index (SMI), and phase angle (PhA) were obtained using a multi-frequency bioelectrical impedance analyzer (S10, InBody, Seoul, Korea).

### 2.4. EEG Recording and Preprocessing

The brain function was assessed by electroencephalogram (EEG) measure of whole brain activity with Quick-20 (Cognionics Inc., San Diego, CA, USA) dry EEG headset. The EEG headset had 19 electrode channels (Fp1, Fp2, F7, F3, Fz, F4, F8, T3, C3, Cz, C4, T4, T5, P3, Pz, P4, T6, O1, and O2) positioned according to the international 10–20 system. The EEG was recorded at a sampling rate of 500 Hz and filtered through a high band-pass of 0.53 and low band-pass of 120 Hz. The electrode impedance was kept under 500 kΩ throughout the recording. During the EEG measurement, participants remained seated with their eyes closed for five min in a dimly lit and quiet room.

EEG noise preprocessing and analyses were conducted using the iSyncBrain^®^ v.2.1.0, 2018 (iMediSync Inc., Seoul, Republic of Korea). A band-pass filter was applied to the EEG data, with the frequency ranging between 1 and 45 Hz. To eliminate noise from the power supplies, a 60 Hz notch filter was also used. Then, the common average reference was applied to remove the noise mixed throughout the recorded EEG data. Artifacts were filtered and removed using bad epoch rejection and independent component analysis to generate clean data for further analysis, i.e., sensor-level and source-level analysis. At sensor level, EEG data was decomposed into different frequency band powers: Delta (1–4 Hz), Theta (4–8 Hz), Alpha (8–12 Hz), Beta (12–30 Hz), and Gamma (30–45 HZ). Absolute power (sum of the component powers for each frequency band) for each frequency band in five brain regions (frontal, temporal, central, parietal, and occipital) were calculated and presented in two-dimensional topographic maps. In the source-level analysis, cortical activity in the brain was analyzed with standardized low-resolution brain electromagnetic tomography technique (sLORETA), which allowed comparison of band powers and functional connectivity in region of interest (ROIs) across the brain. Imaginary coherence (iCoh) was used to estimate functional connectivity. Coherence in EEG has been studied as a measure of brain connectivity [33], and the imaginary part of coherency (iCoh) has been introduced to avoid volume conduction artifacts [34]. We calculated the connectivity in 68 ROIs based on the Desikan–Killiany atlas [35]. The ROIs included were bilateral temporal lobe, frontal lobe, parietal lobe, and occipital lobe. Using the iCoh metrics for each frequency band, we constructed an undirected binary network taking network density into consideration [36]. The measurements of network nodes and edges were defined as ROIs which consisted of node degree, clustering coefficient, characteristic path length, and small-worldness [37]. The characteristic path length was used to measure functional connectivity in this study [38].

### 2.5. Statistical Analyses

Data were analyzed using the IBM Statistical Package for Social Science (SPSS) V23.0 (IBM Corp., Armonk, NY, USA). The baseline mean values for anthropometric, demographic, and physical characteristics were compared with one-way analysis of variance (ANOVA) and the Kruskal–Wallis test for normally and non-normally distributed variables, respectively. Using the intention-to-treat approach, with group × time two-way repeated measures, ANOVA was performed to evaluate differences in physical function measures. Analyses were adjusted for potential covariates such as age and for statistically significant group × time interactions. Statistical analyses for EEG features were assessed using R software (3.5.3) and cloud-based EEG analysis in iSyncBrain^®^. ANOVA was used for the analysis of post-intervention measurement to examine the difference of band powers and functional connectivity between the groups. Bonferroni correction was performed as a post-hoc test. Statistical significance was set at *p* <  0.05.

## 3. Results

The baseline demographic characteristics and physical functions of all participants are described in Table 1. Compared to the RT and the control group, the ERT group was observed to have significantly lower SMI, lower leg muscle mass, and higher grip strength. There were no significant differences in other variables.

**Figure 2:**
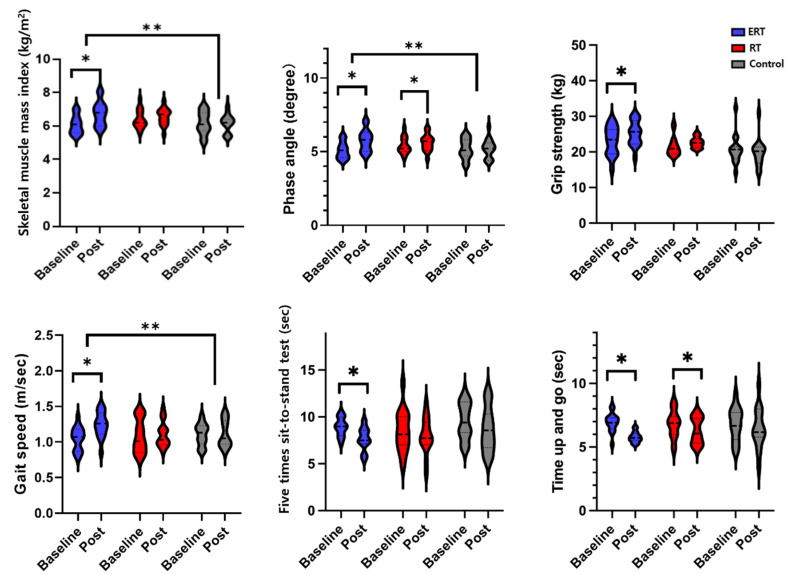
Comparison between baseline and post-intervention for body composition and physical function between ERT, RT, and control groups; ERT, electrical muscle stimulation with resistance exercise training; RT, resistance exercise training; *, between time (*p* < 0.05); **, between groups (*p* < 0.05).

Two-way repeated measures ANOVA revealed a significant main effect on group × time interaction of F (2, 28) = 5.51, *p* < 0.0.1, F (1, 28) = 4.40, *p* < 0.05, and F (2, 28) = 4.00, *p* < 0.05 in SMI, PhA, and gait speed, respectively, at post-intervention in the ERT group compared to the control group as shown in Figure 2. The main effect of time showed statistical significance F (1, 15) = 8.01, *p* < 0.05, and F (1, 14) = 10.8, *p* < 0.05, and F (1, 14) = 6.09, *p* < 0.05 for SMI, PhA, and gait speed, respectively. Within the ERT group, there was increase in SMI from a mean (SD) of 6.0 (1.2) to 6.7 (1.5), phase angle from 5.1 (1.1) to 5.7 (1.9), and gait speed from 1.04 (0.16) to 1.19 (0.18), which corresponded to an effect size of d = 0.25, 0.22, and 0.20, respectively. Within-group analysis of the ERT group showed a significant difference in HGS at post-intervention compared to the baseline. Although repeated measures mixed ANOVA revealed a marginal main effect of group × time interaction, it did not reach statistical significance [F (2, 28) = 2.81, *p* = 0.10]. No significant change was observed in RT and control groups. Conversely, in both ERT and RT groups, FTSS and TUG significantly (*p* < 0.05) decreased. However, repeated measures mixed ANOVA did not show statistically significant main effect of group and group × time interaction [F (2, 26) = 2.19, *p* = 0.13 and F (2, 28) = 1.13, *p* = 0.29].

### 3.1. EEG

#### 3.1.1. Resting State Absolute Band Power

**Figure 3:**
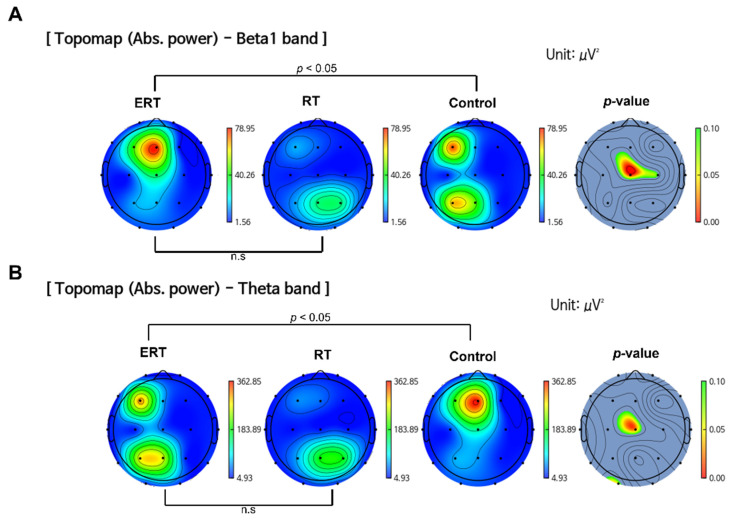
Comparison of band power changes between all groups. (**A**) ERT group showed an increase in the absolute power of Beta1 band compared to RT and control group. (**B**) ERT group showed a decrease in the absolute power of Theta band compared to control group. (The color bars next to ERT, RT, and control group indicate power density: blue indicates lower power density and red indicates higher power density. The color bar next to the *p*-value indicates the *p*-value ranging from 0.10 (green) to 0.00 (red); ERT, electrical muscle stimulation with resistance exercise training; RT, resistance exercise training; n.s, not significant.

The ERT group was observed to have significantly higher Beta1 band power compared to the control group in the central brain region (*p* < 0.05) at post-intervention measurement (Figure 3A). A significant decrease in Theta band power was observed in the ERT group compared to the control group (*p* < 0.05) (Figure 3B). There were no statistically significant differences between the ERT group and RT group. There were no significant changes observed in Delta and Alpha band power.

#### 3.1.2. Resting-State Functional Connectivity

**Figure 4:**
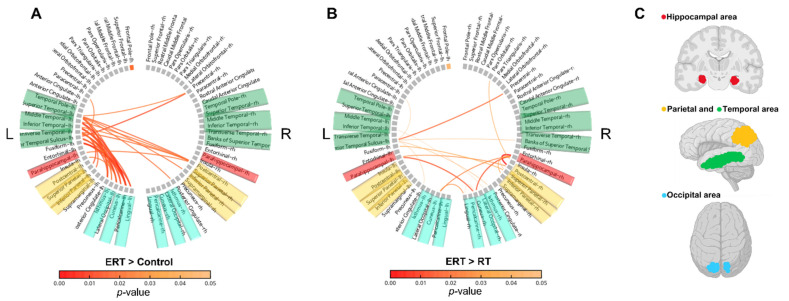
Comparison of changes in the imaginary part of coherence (iCoh) of the Alpha1 band and corresponding visual coordinates. The red lines represent a significant increase in the iCoh of the Alpha1 band. The iCoh is the estimated functional connectivity. (**A**) Comparison of ERT group compared to control group. (**B**) Comparison of ERT group compared to RT group. (**C**) Visual coordinates of brain areas corresponding to A and B; ERT, electrical muscle stimulation with resistance exercise training; RT, resistance exercise training.

At resting state, alpha waves are predominantly present in the brain. In the ERT group compared to the control group, Alpha1 band connectivity was found to be increased significantly (*p* < 0.05) (Figure 4A). The increased connectivity is represented with red lines. The areas with increased connection density were the temporal, hippocampal, parietal, and occipital areas. Similarly, the ERT group also had higher connectivity compared to the RT group (*p* < 0.05) (Figure 4B). The connectivity density was observed to be more prominent in the hippocampal, parietal, and occipital areas. The brain areas with high connection density are visually represented as colored areas (Figure 4C), where red, yellow, green, and blue represent the hippocampal, parietal, temporal, and occipital regions, respectively. 

## 4. Discussion

To our knowledge, no prior studies have examined the effect of combined ERT and single-bout of RT on physical and brain function in middle-aged and older female population. We observed significant improvement of ERT on physical functions such as SMI, PhA and gait speed compared to control group. Furthermore, compared to control, ERT showed considerable enhancement in brain activity as shown by increased beta1 band power, decreased theta band power and increased al-pha1 connectivity. However, in RT group, although there were changes in PhA and TUG at the post intervention measurement compared to baseline, RT did not show significant improvements in physical and brain function compared to control group.

A well-established connection has been demonstrated between muscle strength and quantity for good health [1]. In order to increase muscle, RT has been widely proven to be an effective method [16,39] with intervention duration ranging from 8 weeks [40,41] to 12 weeks [42,43]. Similarly, in order to maintain muscle mass and function, EMS has been used for more than a decade in the population of those who cannot perform exercise due to barriers such as disease and/or disability [44]. When EMS is combined with RT, we observed a significant increase in skeletal muscle mass even with a short duration of training (i.e., four weeks) in our study. We also observed a significant increase in PhA in both ERT and RT groups. RT has also been reported to increase PhA [45], which is considered to be a sign of greater muscle mass as well as strength [46]. A direct relationship has been observed between PhA and the volume of intracellular fluids, which increases in relation to muscle tissue, resulting in higher PhA in those with greater muscle quantity [46].

Another physical parameter commonly associated with muscle mass is grip strength [47], which improved significantly in the ERT group. The increase in skeletal muscle mass might have led to an increase in muscle strength. RT increases skeletal muscle synthesis proteins and decreases catabolic level [48,49], leading to enhanced muscular strength. We also observed improvements in gait speed, TUG, and FTSS in the ERT group. One common thing about these tests is that they require the use of the lower limbs. In our study, the exercises contents were mostly at the lower limbs (quadriceps), which plays a crucial role in maintaining functional mobility as well as increasing gait speed. Moreover, EMS has also been reported to directly stimulate protein synthesis rates of skeletal muscle [50]. Therefore, the increase in skeletal muscle mass and muscle strength by ERT intervention may have resulted in improved functional mobility leading to improved gait speed, TUG, and FTSS in our study.

Previous studies on RT [51,52] and EMS [29] have been reported to increase BDNF concentration. BDNF plays an important role in synaptic plasticity [53], which is related to the activity-related changes in neurons, which are associated with learning and memory [54]. The proposed mechanism through which exercise induces BDNF concentration is through the induction of expression of Fndc5 [55], which is a PGC-1α-dependent myokine, in skeletal muscle after exercise stimulation [56]. As for EMS, increase in sympathetic nerve activity might have contributed to increase in BDNF. Transcutaneous nerve stimulation has been reported to enhance sympathetic nerve activity in humans through increased sensation in skin and muscle contraction [57,58]. Previous animal studies have revealed production of BDNF with increase in sympathetic nerve activity in the adrenal medulla through the sympathetic–adrenal–medullary axis [59]. Additionally, EMS might have contributed to increased BDNF through lactate production. Lactate is considered as a key component for the enhancement of peripheral BDNF [60,61]. EMS administration to muscle activates both slow- and fast-twitch muscle fibers [62] and fast-twitch fibers have been recognized as major physiological lactate producers [63]. The increased lactate through use of EMS may have led to BDNF production. 

Therefore, in this study, in addition to physical benefits, we also observed enhanced brain activity in the central, temporal, parietal, and hippocampal brain regions in the ERT group indicated by changes in Beta1, Theta, and Alpha1 power band. Decrease in Beta power and increase in Theta power are considered to be the earliest changes to occur in cognitive decline [64]. Beta band power, especially in the parietal region, has been associated to have negative correlation with amyloid deposition and positive correlation with anterograde memory in MCI [65]. Our study also found significant results in Alpha1 band connectivity, the default resting-state oscillating rhythm, in hippocampal, temporal, and parietal brain areas. A decrease in alpha connectivity is reported to be associated with lower cognitive scores [66], atrophy of the hippocampus [67], and amyloid deposition in the brain [68]. As BDNF has also been associated with change in hippocampal volume [53,69], this might explain the positive changes observed in that particular region in our study. This positive improvement in the brain might also add to improved gait speed observed in our study, as slow gait is believed to be related to different underlying mechanisms, such as neurodegeneration and inflammation [27,70]. On the other hand, we did not observe any significant changes in brain function in the RT group which might be due to the shorter duration of intervention. 

Likewise, the ERT group showed significant changes in all physical function measures, whereas the RT group showed improvements in some measures of physical functions such as TUG and PhA. Therefore, the single-bout exercise period may have been too short to show changes in physical function. This aspect of our study shed light to the fact that EMS might have escalated the benefits of RT leading to improvement in physical and brain function. The strength of our study includes the use of portable EMS devices which increased the feasibility. Moreover, we used EEG to observe the effectiveness of intervention at the functional level. Although the results are optimistic, there were two limitations. This study sample was small and homogenous (only female participants). Future studies should consider a large sample size including both male and female populations and RT of varying intensity. In addition, administering more measures of cognitive markers might be helpful to provide additional insight on the effect of ERT on brain health.

## 5. Conclusions

The present findings showed that a four-week ERT significantly improves SMI, PhA, gait speed, and brain function in middle-aged and older women, whereas RT did not show significant improvements compared to the control group. Despite shorter duration, ERT intervention had benefits on both physical and brain function compared to RT. Therefore, ERT might also be beneficial for individuals with sarcopenic and cognitive frailty to improve muscle loss and brain function and be a feasible alternative strategy to single-bout RT to prevent sarcopenia and frailty in the community. Further studies with larger sample sizes and longer follow-up periods are required to confirm the findings of our preliminary study.

## Figures and Tables

**Table 1:** Baseline characteristics of all participants by group.

Variable	Total	ERT	RT	Control	*p*-Value
	(*n* = 48)	(*n* = 16)	(*n* = 16)	(*n* = 16)	
Age (years)	69.1 ± 5.3	69.2 ± 4.0	69.4 ± 5.2	68.6 ± 7.1	0.96
Height (cm)	154.5 ± 4.4	154.8 ± 4.3	154.5 ± 4.5	154.2 ± 4.7	0.90
Weight (kg)	58.4 ± 8.1	59.5 ± 11.3	57.6 ± 5.6	57.8 ± 5.7	0.99
BMI (kg/m^2^)	24.4 ± 2.8	24.1 ± 3.6	24.7 ± 2.6	24.4 ± 1.9	0.68
SMI (kg/m^2^)	6.2 ± 0.9	6.1 ± 1.0	6.4 ± 1.0	6.1 ± 1.0	0.60
Phase angle (°)	5.3 ± 1.0	5.1 ± 1.1	5.6 ± 1.0	5.2 ± 1.0	0.38
Grip strength (kg)	22.7 ± 4.2	23.8 ± 4.1	22.8 ± 3.2	21.3 ± 5.1	0.24
Gait speed (m/s)	1.08 ± 0.18	1.04 ± 0.16	1.12 ± 0.23	1.08 ± 0.16	0.55
FSST (s)	8.9 ± 1.8	8.9 ± 0.9	8.2 ± 2.3	9.6 ± 1.8	0.16
TUG (s)	6.7 ± 1.1	6.9 ± 0.7	6.6 ± 1.1	6.7 ± 1.4	0.75

Values are expressed in mean ± standard deviation; ERT, electrical muscle stimulation with resistance exercise training; RT, resistance exercise training; BMI, body mass index; SMI, skeletal muscle mass index; FTSS, five times sit-to-stand; TUG, timed up-and-go.

## Data Availability

Qualified researchers can obtain the data from the corresponding author (htpark@dau.ac.kr). The data are not publicly available due to privacy concerns imposed by the IRB.

## References

[1] Cruz-Jentoft A.J., Bahat G., Bauer J., Boirie Y., Bruyère O., Cederholm T., Cooper C., Landi F., Rolland Y., Sayer A.A. (2019). Sarcopenia: Revised European consensus on definition and diagnosis. Age Ageing.

[2] Delmonico M.J., Harris T.B., Lee J.S., Visser M., Nevitt M., Kritchevsky S.B., Tylavsky F.A., Newman A.B., Health A., Study B.C. (2007). Alternative definitions of sarcopenia, lower extremity performance, and functional impairment with aging in older men and women. J. Am. Geriatr. Soc..

[3] Goodpaster B.H., Park S.W., Harris T.B., Kritchevsky S.B., Nevitt M., Schwartz A.V., Simonsick E.M., Tylavsky F.A., Visser M., Newman A.B. (2006). The loss of skeletal muscle strength, mass, and quality in older adults: The health, aging and body composition study. J. Gerontol. Ser. A Biol. Sci. Med. Sci..

[4] Sousa-Victor P., Muñoz-Cánoves P. (2016). Regenerative decline of stem cells in sarcopenia. Mol. Asp. Med..

[5] Santilli V., Bernetti A., Mangone M., Paoloni M. (2014). Clinical definition of sarcopenia. Clin. Cases Miner. Bone Metab..

[6] Aprahamian I., Cipolli G.C., Yassuda M.S. (2020). Sarcopenia and cognitive impairment: Possible physiopathological causation or just a spurious association?. Clin. Nutr..

[7] Beeri M.S., Leugrans S.E., Delbono O., Bennett D.A., Buchman A.S. (2021). Sarcopenia is associated with incident Alzheimer’s dementia, m ild cognitive impairment, and cognitive decline. J. Am. Geriatr. Soc..

[8] Wallace L.M., Theou O., Godin J., Andrew M.K., Bennett D.A., Rockwood K. (2019). Investigation of frailty as a moderator of the relationship between neuropathology and dementia in Alzheimer’s disease: A cross-sectional analysis of data from the Rush Memory and Aging Project. Lancet Neurol..

[9] Yu L., Boyle P.A., Leurgans S.E., Wilson R.S., Bennett D.A., Buchman A.S. (2019). Incident mobility disability, mild cognitive impairment, and mortality in community-dwelling older adults. Neuroepidemiology.

[10] Kim H., Hirano H., Edahiro A., Ohara Y., Watanabe Y., Kojima N., Kim M., Hosoi E., Yoshida Y., Yoshida H. (2016). Sarcopenia: Prevalence and associated factors based on different suggested definitions in community-dwelling older adults. Geriatr. Gerontol. Int..

[11] Sayer A.A., Robinson S.M., Patel H.P., Shavlakadze T., Cooper C., Grounds M.D. (2013). New horizons in the pathogenesis, diagnosis and management of sarcopenia. Age Ageing.

[12] Lopez O.L., Jagust W.J., Dulberg C., Becker J.T., DeKosky S.T., Fitzpatrick A., Breitner J., Lyketsos C., Jones B., Kawas C. (2003). Risk factors for mild cognitive impairment in the Cardiovascular Health Study Cognition Study: Part 2. Arch Neurol-Chic..

[13] Wang L., Larson E.B., Bowen J.D., van Belle G. (2006). Performance-based physical function and future dementia in older people. Arch. Intern. Med..

[14] Broadhouse K.M., Singh M.F., Suo C., Gates N., Wen W., Brodaty H., Jain N., Wilson G.C., Meiklejohn J., Singh N. (2020). Hippocampal plasticity underpins long-term cognitive gains from resistance exercise in MCI. NeuroImage Clin..

[15] Dent E., Morley J., Cruz-Jentoft A., Arai H., Kritchevsky S., Guralnik J., Bauer J., Pahor M., Clark B., Cesari M. (2018). International clinical practice guidelines for sarcopenia (ICFSR): Screening, diagnosis and management. J. Nutr. Health Aging.

[16] Liu C.-j., Latham N.K. (2009). Progressive resistance strength training for improving physical function in older adults. Cochrane Database Syst. Rev..

[17] Grgic J., Garofolini A., Orazem J., Sabol F., Schoenfeld B.J., Pedisic Z. (2020). Effects of resistance training on muscle size and strength in very elderly adults: A systematic review and meta-analysis of randomized controlled trials. Sport. Med..

[18] Straight C.R., Lindheimer J.B., Brady A.O., Dishman R.K., Evans E.M. (2016). Effects of resistance training on lower-extremity muscle power in middle-aged and older adults: A systematic review and meta-analysis of randomized controlled trials. Sport. Med..

[19] Pahor M., Guralnik J.M., Ambrosius W.T., Blair S., Bonds D.E., Church T.S., Espeland M.A., Fielding R.A., Gill T.M., Groessl E.J. (2014). Effect of structured physical activity on prevention of major mobility disability in older adults: The LIFE study randomized clinical trial. Jama.

[20] Hillman C.H., Erickson K.I., Kramer A.F. (2008). Be smart, exercise your heart: Exercise effects on brain and cognition. Nat. Rev. Neurosci..

[21] Hurst C., Robinson S.M., Witham M.D., Dodds R.M., Granic A., Buckland C., De Biase S., Finnegan S., Rochester L., Skelton D.A. (2022). Resistance exercise as a treatment for sarcopenia: Prescription and delivery. Age Ageing.

[22] Karatzanos E., Gerovasili V., Zervakis D., Tripodaki E.-S., Apostolou K., Vasileiadis I., Papadopoulos E., Mitsiou G., Tsimpouki D., Routsi C. (2012). Electrical muscle stimulation: An effective form of exercise and early mobilization to preserve muscle strength in critically ill patients. Crit. Care Res. Pract..

[23] Zanotti E., Felicetti G., Maini M., Fracchia C. (2003). Peripheral muscle strength training in bed-bound patients with COPD receiving mechanical ventilation: Effect of electrical stimulation. Chest.

[24] Vivodtzev I., Pépin J.-L., Vottero G., Mayer V., Porsin B., Lévy P., Wuyam B. (2006). Improvement in quadriceps strength and dyspnea in daily tasks after 1 month of electrical stimulation in severely deconditioned and malnourished COPD. Chest.

[25] Nuhr M.J., Pette D., Berger R., Quittan M., Crevenna R., Huelsman M., Wiesinger G.F., Moser P., Fialka-Moser V., Pacher R. (2004). Beneficial effects of chronic low-frequency stimulation of thigh muscles in patients with advanced chronic heart failure. Eur. Heart J..

[26] Banerjee P., Caulfield B., Crowe L., Clark A. (2005). Prolonged electrical muscle stimulation exercise improves strength and aerobic capacity in healthy sedentary adults. J. Appl. Physiol..

[27] Kern H., Barberi L., Löfler S., Sbardella S., Burggraf S., Fruhmann H., Carraro U., Mosole S., Sarabon N., Vogelauer M. (2014). Electrical stimulation counteracts muscle decline in seniors. Front. Aging Neurosci..

[28] Rahmati M., Gondin J., Malakoutinia F. (2021). Effects of Neuromuscular Electrical Stimulation on Quadriceps Muscle Strength and Mass in Healthy Young and Older Adults: A Scoping Review. Phys. Ther..

[29] Nishikawa Y., Watanabe K., Kawade S., Takahashi T., Kimura H., Maruyama H., Hyngstrom A. (2019). The effect of a portable electrical muscle stimulation device at home on muscle strength and activation patterns in locomotive syndrome patients: A randomized control trial. J. Electromyogr. Kinesiol..

[30] Hasegawa S., Kobayashi M., Arai R., Tamaki A., Nakamura T., Moritani T. (2011). Effect of early implementation of electrical muscle stimulation to prevent muscle atrophy and weakness in patients after anterior cruciate ligament reconstruction. J. Electromyogr. Kinesiol..

[31] Faul F., Erdfelder E., Lang A.-G., Buchner A. (2007). G* Power 3: A flexible statistical power analysis program for the social, behavioral, and biomedical sciences. Behav. Res. Methods.

[32] Banerjee P., Caulfield B., Crowe L., Clark A.L. (2009). Prolonged electrical muscle stimulation exercise improves strength, peak VO_2_, and exercise capacity in patients with stable chronic heart failure. J. Card. Fail..

[33] Nunez P.L., Srinivasan R., Westdorp A.F., Wijesinghe R.S., Tucker D.M., Silberstein R.B., Cadusch P.J. (1997). EEG coherency: I: Statistics, reference electrode, volume conduction, Laplacians, cortical imaging, and interpretation at multiple scales. Electroencephalogr. Clin. Neurophysiol..

[34] Nolte G., Bai O., Wheaton L., Mari Z., Vorbach S., Hallett M. (2004). Identifying true brain interaction from EEG data using the imaginary part of coherency. Clin. Neurophysiol..

[35] Desikan R.S., Ségonne F., Fischl B., Quinn B.T., Dickerson B.C., Blacker D., Buckner R.L., Dale A.M., Maguire R.P., Hyman B.T. (2006). An automated labeling system for subdividing the human cerebral cortex on MRI scans into gyral based regions of interest. Neuroimage.

[36] Hassan M., Dufor O., Merlet I., Berrou C., Wendling F. (2014). EEG source connectivity analysis: From dense array recordings to brain networks. PLoS ONE.

[37] Xia M., Wang J., He Y. (2013). BrainNet Viewer: A network visualization tool for human brain connectomics. PLoS ONE.

[38] Rubinov M., Sporns O. (2010). Complex network measures of brain connectivity: Uses and interpretations. Neuroimage.

[39] Melov S., Tarnopolsky M.A., Beckman K., Felkey K., Hubbard A. (2007). Resistance exercise reverses aging in human skeletal muscle. PLoS ONE.

[40] Henwood T.R., Taaffe D.R. (2005). Improved physical performance in older adults undertaking a short-term programme of high-velocity resistance training. Gerontology.

[41] Baker B.S., Weitzel K.J., Royse L.A., Miller K., Guess T.M., Ball S.D., Duren D.L. (2020). Efficacy of an 8-week resistance training program in Older adults: A randomized controlled trial. J. Aging Phys. Act..

[42] Sayers S.P., Gibson K. (2010). A comparison of high-speed power training and traditional slow-speed resistance training in older men and women. J. Strength Cond. Res..

[43] Skelton D.A., Young A., Greig C.A., Malbut K.E. (1995). Effects of resistance training on strength, power, and selected functional abilities of women aged 75 and older. J. Am. Geriatr. Soc..

[44] Maffiuletti N.A., Gondin J., Place N., Stevens-Lapsley J., Vivodtzev I., Minetto M.A. (2018). Clinical use of neuromuscular electrical stimulation for neuromuscular rehabilitation: What are we overlooking?. Arch. Phys. Med. Rehabil..

[45] Dos Santos L., Cyrino E., Antunes M., Santos D., Sardinha L. (2016). Changes in phase angle and body composition induced by resistance training in older women. Eur. J. Clin. Nutr..

[46] Matias C.N., Campa F., Nunes C.L., Francisco R., Jesus F., Cardoso M., Valamatos M.J., Homens P.M., Sardinha L.B., Martins P. (2021). Phase Angle Is a Marker of Muscle Quantity and Strength in Overweight/Obese Former Athletes. Int. J. Environ. Res. Public Health.

[47] Chan J., Lu Y.-C., Yao M.M.-S., Kosik R.O. (2022). Correlation between hand grip strength and regional muscle mass in older Asian adults: An observational study. BMC Geriatr..

[48] Peterson M.D., Rhea M.R., Sen A., Gordon P.M. (2010). Resistance exercise for muscular strength in older adults: A meta-analysis. Ageing Res. Rev..

[49] Agergaard J., Bülow J., Jensen J.K., Reitelseder S., Drummond M.J., Schjerling P., Scheike T., Serena A., Holm L. (2017). Light-load resistance exercise increases muscle protein synthesis and hypertrophy signaling in elderly men. Am. J. Physiol. Endocrinol. Metab..

[50] Wall B.T., Dirks M.L., Verdijk L.B., Snijders T., Hansen D., Vranckx P., Burd N.A., Dendale P., Van Loon L.J. (2012). Neuromuscular electrical stimulation increases muscle protein synthesis in elderly type 2 diabetic men. Am. J. Physiol. Endocrinol. Metab..

[51] Vega S.R., Knicker A., Hollmann W., Bloch W., Strüder H. (2010). Effect of resistance exercise on serum levels of growth factors in humans. Horm. Metab. Res..

[52] Yarrow J.F., White L.J., McCoy S.C., Borst S.E. (2010). Training augments resistance exercise induced elevation of circulating brain derived neurotrophic factor (BDNF). Neurosci. Lett..

[53] Leal G., Afonso P.M., Salazar I.L., Duarte C.B. (2015). Regulation of hippocampal synaptic plasticity by BDNF. Brain Res..

[54] Magee J.C., Grienberger C. (2020). Synaptic plasticity forms and functions. Annu. Rev. Neurosci..

[55] Wrann C.D., White J.P., Salogiannnis J., Laznik-Bogoslavski D., Wu J., Ma D., Lin J.D., Greenberg M.E., Spiegelman B.M. (2013). Exercise induces hippocampal BDNF through a PGC-1α/FNDC5 pathway. Cell Metab..

[56] Timmons J.A., Baar K., Davidsen P.K., Atherton P.J. (2012). Is irisin a human exercise gene?. Nature.

[57] Moreau D., Dubots P., Boggio V., Guilland J.C., Cometti G. (1995). Effects of electromyostimulation and strength training on muscle soreness, muscle damage and sympathetic activation. J. Sport. Sci..

[58] Wong R.A., Jette D.U. (1984). Changes in sympathetic tone associated with different forms of transcutaneous electrical nerve stimulation in healthy subjects. Phys. Ther..

[59] Kondo Y., To M., Saruta J., Hayashi T., Sugiyama H., Tsukinoki K. (2013). Role of TrkB expression in rat adrenal gland during acute immobilization stress. J. Neurochem..

[60] Schiffer T., Schulte S., Sperlich B., Achtzehn S., Fricke H., Strüder H.K. (2011). Lactate infusion at rest increases BDNF blood concentration in humans. Neurosci. Lett..

[61] Ferris L.T., Williams J.S., Shen C.-L. (2007). The effect of acute exercise on serum brain-derived neurotrophic factor levels and cognitive function. Med. Sci. Sport. Exerc..

[62] Maffiuletti N.A., Minetto M.A., Farina D., Bottinelli R. (2011). Electrical Stimulation for Neuromuscular Testing and Training: State-of-the Art and Unresolved Issues.

[63] Philp A., Macdonald A.L., Watt P.W. (2005). Lactate—A signal coordinating cell and systemic function. J. Exp. Biol..

[64] Han S.-H., Pyun J.-M., Yeo S., Kang D.W., Jeong H.T., Kang S.W., Kim S., Youn Y.C. (2021). Differences between memory encoding and retrieval failure in mild cognitive impairment: Results from quantitative electroencephalography and magnetic resonance volumetry. Alzheimer’s Res. Ther..

[65] Musaeus C.S., Nielsen M.S., Østerbye N.N., Høgh P. (2018). Decreased parietal beta power as a sign of disease progression in patients with mild cognitive impairment. J. Alzheimer’s Dis..

[66] Babiloni C., Binetti G., Cassetta E., Dal Forno G., Del Percio C., Ferreri F., Ferri R., Frisoni G., Hirata K., Lanuzza B. (2006). Sources of cortical rhythms change as a function of cognitive impairment in pathological aging: A multicenter study. Clin. Neurophysiol..

[67] Babiloni C., Frisoni G.B., Pievani M., Vecchio F., Lizio R., Buttiglione M., Geroldi C., Fracassi C., Eusebi F., Ferri R. (2009). Hippocampal volume and cortical sources of EEG alpha rhythms in mild cognitive impairment and Alzheimer disease. Neuroimage.

[68] Michels L., Muthuraman M., Anwar A.R., Kollias S., Leh S.E., Riese F., Unschuld P.G., Siniatchkin M., Gietl A.F., Hock C. (2017). Changes of functional and directed resting-state connectivity are associated with neuronal oscillations, ApoE genotype and amyloid deposition in mild cognitive impairment. Front. Aging Neurosci..

[69] Erickson K.I., Miller D.L., Roecklein K.A. (2012). The aging hippocampus: Interactions between exercise, depression, and BDNF. Neuroscientist.

[70] Quan M., Xun P., Chen C., Wen J., Wang Y., Wang R., Chen P., He K. (2017). Walking pace and the risk of cognitive decline and dementia in elderly populations: A meta-analysis of prospective cohort studies. J. Gerontol. Ser. A.

